# Domestic Medicine

**Published:** 1872-04

**Authors:** 


### Domestic Medicine.

### INFORMATION FOR THE HOUSEHOLD.

PREPARED BY THE EDITOR.

COUGH MIXTURE.—Take hydrochlorate of morphia, one-half grain; glycerine, two fluid ounces. Mix. Dose: A teaspoonful when the cough is troublesome.

---

BRUISES.—Apply repeatedly cloths wrung out of hot water, and bathe the parts in tincture of arnica. This will prevent discoloration of the skin, or a "black eye," if applied immediately.

---

TO STOP BLEEDING FROM LEECH BITE.—Make a ball of cotton about the size of a pea; put this pellet of cotton or lint upon the wound; press it down firmly; keep up the pressure for a quarter of an hour; remove the finger cautiously, taking care to let the pellet remain.

---

TO BRIGHTEN STRAW MATTING AND OIL-CLOTH.—Give the matting a thorough washing with salt and water, using one pint of salt in a pail of water. Dry quickly with a soft cloth. Rub your oil cloth with a cloth dipped in milk, first having scrubbed it with soap and warm water. It will then look as bright as when new.

---

TO REMOVE PAINT FROM STONE OR WOOD.—Dissolve three pounds of washing soda in a gallon of boiling water. Saturate the surface to be cleaned with this preparation, allowing it to remain 20 minutes. At the expiration of this time the paint can be washed off with a cloth dipped in hot water.

---

FLIES.—Time will soon be here when we will be annoyed by house flies. They may be captured and killed by thousands, in the following manner: Boil together equal parts, by weight, of glue and molasses, spread it over common brown paper, while hot, with a brush. Place a sheet of the paper in every room in your house. It will capture every fly in the room within the day. The paper can be thrown in the fire and a new one used, when covered with the captured flies.

---

TO DISGUISE CASTOR OIL.—Rub up two drops of oil of cinnamon with an ounce of glycerine, and add an ounce of castor oil.—Children will take it as a luxury and ask for more.

To PREVENT IRON RUST.—Kerosene applied to stoves or farming implements, during summer, will prevent their rusting.

TOOTH-ACHE.—Introduce into the cavity of the tooth as much *chloral hydrate* as it will contain. It will arrest all pain instantly.

To CLEAN SILVER.—Wash in hot soap suds, then clean with a paste made of whiting and alcohol. Polish with buckskin.

CHILBLAINS.—Tincture chloride of iron applied to chilblains or frost-bites, by means of a brush, morning and evening, is an unfailing cure.

ITCH.—Rub the parts affected with common benzine. Then take a hot bath for 30 minutes, after which apply the benzine again.—This is more than the itch-insect can stand—he'll curl up and die instanter.

FOR CHAFES.—The *Pacific Medical & Surgical Journal* recommends two parts of powdered soapstone and one part of calomel, as the most elegant and effective dry application to the chafed skin of infants.

SUBSTITUTE FOR CREAM.—Beat up a fresh egg in a basin, and then pour boiling tea over it gradually, to prevent its curdling. It is difficult from the taste to distinguish the composition from cream. Shut your eyes and try it

MARKING INK.—Dissolve asphaltum, grahamite, albertite, or any mineral of this character, in naptha or oil of turpentine to a thin fluid. This will form an ink that will dry quickly, will not spread, and the markings are nearly indestructable. It is intended for marking packages with a brush.

BURNS AND SCALDS.—Every family should have a preparation of flax-seed oil, chalk and vinegar, about the consistency of thick paint, constantly on hand for burns and scalds. A noted physician stated that he has employed the mixture extensively in hospital and private practice for the past forty years, and believes that no application can compare with it, as regards relief of pain and curative results.

To RESTORE FURNITURE.—An old cabinet maker says the best preparation for cleaning furniture that is marred or scratched, is a mixture of three parts of linseed-oil and one part spirits of turpentine. It not only covers the disfigured surface, but restores wood to its original color, leaving a lustre upon its surface. Put on with a woollen cloth, and when dry, rub with woollen.

To WHITEN STRAW HATS.—Get out your last summer's straw hats and be your own bleacher. You can make them as white as when new, in this manner: Scrape stick sulphur with a knife, mix the powder to a mush with water; plaster this over your straw, and place in hot water several hours. Brush off when dry. This is an easy and effectual plan.

"COLD IN THE HEAD."—When the head feels swollen, the nose obstructed, the forehead hot, and the eyes watery; snuff a few drops of tincture of camphor up the nose, every hour or two, and take internally five or six drops on a lump of sugar. Ordinary cold, and even influenza, if treated in this manner, at the *very beginning* of the attack, is generally controlled at once.

POISON BY IVY.—Dr. J. D. Stewart, of Coila, N. Y., writes to the *Boston Journal of Chemistry*, that he has used the following remedy in poisoning by ivy, which at once arrests the tormenting burning pain, and cures the worst cases in one or two days:

Bruise, slightly, a handful of white oak leaves; add new milk enough to cover; simmer ten minutes, and apply *as hot as can be borne*, three times a day.

DYSENTERY.—The following simple remedy has been known to cure the most obstinate cases of dysentery, when other remedies had failed. It has the merit of being harmless and almost always effectual: Take one-fourth of a pint of hot water; vinegar, half a pint. Mix. Now add common salt as long as it will dissolve in the mixture, stirring it freely. Give for an adult one table-spoonful every hour, until the bloody discharges cease, or until it operates freely upon the bowels.

